# When Artificial Intelligence (AI) Met a Botfly: The First AI-Assisted Diagnosis of Cutaneous Furuncular Myiasis in a Community-Based Surgical Practice

**DOI:** 10.7759/cureus.77629

**Published:** 2025-01-18

**Authors:** Joseph P Maurice, David Santos

**Affiliations:** 1 Surgery, St. Anne Hospital, Burien, USA; 2 Biological Sciences, University of California Berkeley, Berkeley, USA

**Keywords:** artificial intelligence in surgery, botfly, dermatobia hominis, furuncular myiasis, human botfly

## Abstract

In this report, we describe a rare case of a parasitic infection of *Dermatobia hominis* in a patient who presented to a general otolaryngology clinic in a Seattle suburb. The 69-year-old male patient initially presented with a unique furuncle on his scalp after returning from a vacation in Belize. Initially unrecognized as a cutaneous manifestation of *D. hominis *larva, the patient was brought for surgical excision and biopsy of the lesion to a community-based surgical center. This case highlights how artificial intelligence (AI) was innovatively utilized intraoperatively to assist the surgeon in confirming the diagnosis of this rare infection and facilitating treatment. AI has rapidly become an important tool used by physicians worldwide and is evolving in its applications in healthcare. AI-assisted image analysis has recently been demonstrated to be effective in assisting in the diagnosis and treatment of skin conditions including cancers and rare parasitic conditions such as leishmaniasis. A review of the literature reveals that our report is a first on AI-assisted intraoperative diagnosis of *D. hominis*. We discuss this case, the differential diagnosis, treatment options, and innovative use of ubiquitous AI in the operating room to efficiently assist clinicians in confirming their suspicions and ensuring appropriate treatment.

## Introduction

The introduction of artificial intelligence (AI) in community-based medical care is rapidly transforming healthcare. AI systems, in these settings, are revolutionizing clinical decision-making, facilitating charting, assisting patient-physician relationships, and optimizing resource allocation [1].

Medical imaging innovation utilizing AI has begun to improve diagnostic accuracy and treatment modalities [2]. The use of AI to assist in the recognition of rare diseases has also become a burgeoning research field [3]. Rare diseases are often misdiagnosed, posing a challenge for appropriate management, especially in rural community-based practices that may have limited resources or availability of medical expertise. A common goal among those working with AI applications in medicine and rare diseases is to accurately assist in the diagnosis and, consequently, aid in the appropriate management of these conditions. For example, leishmaniasis, the second most deadly parasitic disease in the world, presents a challenge to clinicians seeking to diagnose it efficiently and accurately. Often due to its predominance in Third World countries, clinicians worldwide have struggled with the diagnosis and treatment of leishmaniasis, largely owing to limited pathology resources such as insufficient laboratory infrastructure and the technical, time-consuming expertise needed for diagnosis (i.e., Giemsa staining, cultures, and polymerase chain reaction (PCR)-based methods).

Recent developments in rapid automated AI image analysis of microscopic leishmaniasis specimens have shown to be an efficient alternative to assist in the diagnosis of this parasitic disease. Broadly available AI technologies offer innovative solutions that can improve conventional patient care worldwide. Globally, specialized pathologists and the tertiary care infrastructure surrounding these specialists are not always immediately accessible or are cost-prohibitive in common surgical theaters (i.e., office-based practices, ambulatory surgery centers, and rural hospitals). The ubiquitous and inexpensive presence of AI-based image analysis can offer an untapped solution for these care centers with limited resources, as demonstrated by the AI-assisted benefits found in the diagnosis and care of other rare diseases.

The rapidly evolving synthesis of AI technologies with dermatologic and infectious disease diagnoses and management presents an important, optimistic trajectory toward overcoming these clinical challenges and advancing quality care, especially in the broader landscape of underserved rural community medicine and global health.

## Case presentation

The patient, a 69-year-old male, presented to a general suburban otolaryngology clinic with a chief complaint of a right post-auricular scalp lesion. The patient recalled receiving several mosquito bites to his head, neck, and extremities four weeks prior while on vacation in the rainforests of Belize. He perceived this post-auricular scalp wound as a bite that had failed to heal and said that the area had become more intensely pruritic and mildly painful over the last several days. The wound had started to drain clear fluid, staining his pillow at night, since he returned to the United States. He denied any fever, sweats, chills, diarrhea, nausea, coughing or vomiting. Additionally, he denied a prior history of sebaceous cysts or skin cancers. 

**Figure 1:**
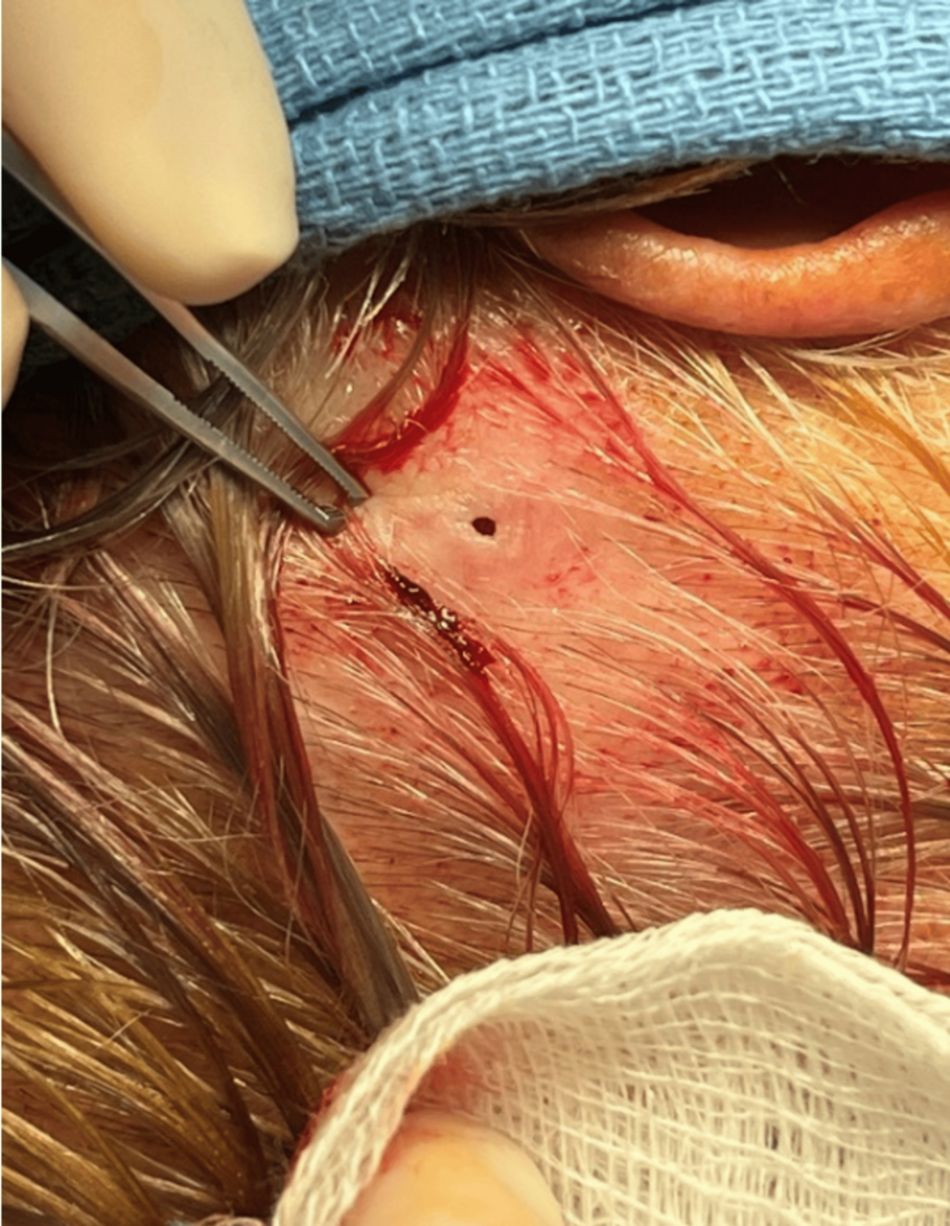
Furuncular lesion of Dermatobia hominis. The 1 mm opening allowed the larva to breathe and is a hallmark of this parasitic infection.

Examination of the scalp revealed a slightly raised 2 cm x 2 cm, soft, tender-to-palpation, non-fluctuant lesion within the hair-bearing scalp, as shown in Figure 1. Centrally, within the lesion, there was a 1 mm well-circumscribed opening that was draining scant odorless serous fluid. Under binocular microscopy, no evidence of a foreign body within the lesion or punctum was observed, nor was there any purulence or surrounding erythema. The differential diagnosis at this juncture included scalp cellulitis/folliculitis, allergic reaction to an insect bite, a foreign body, an evolving scalp abscess or furunculosis, an infected or ruptured sebaceous cyst, or neoplasm. 

**Figure 2:**
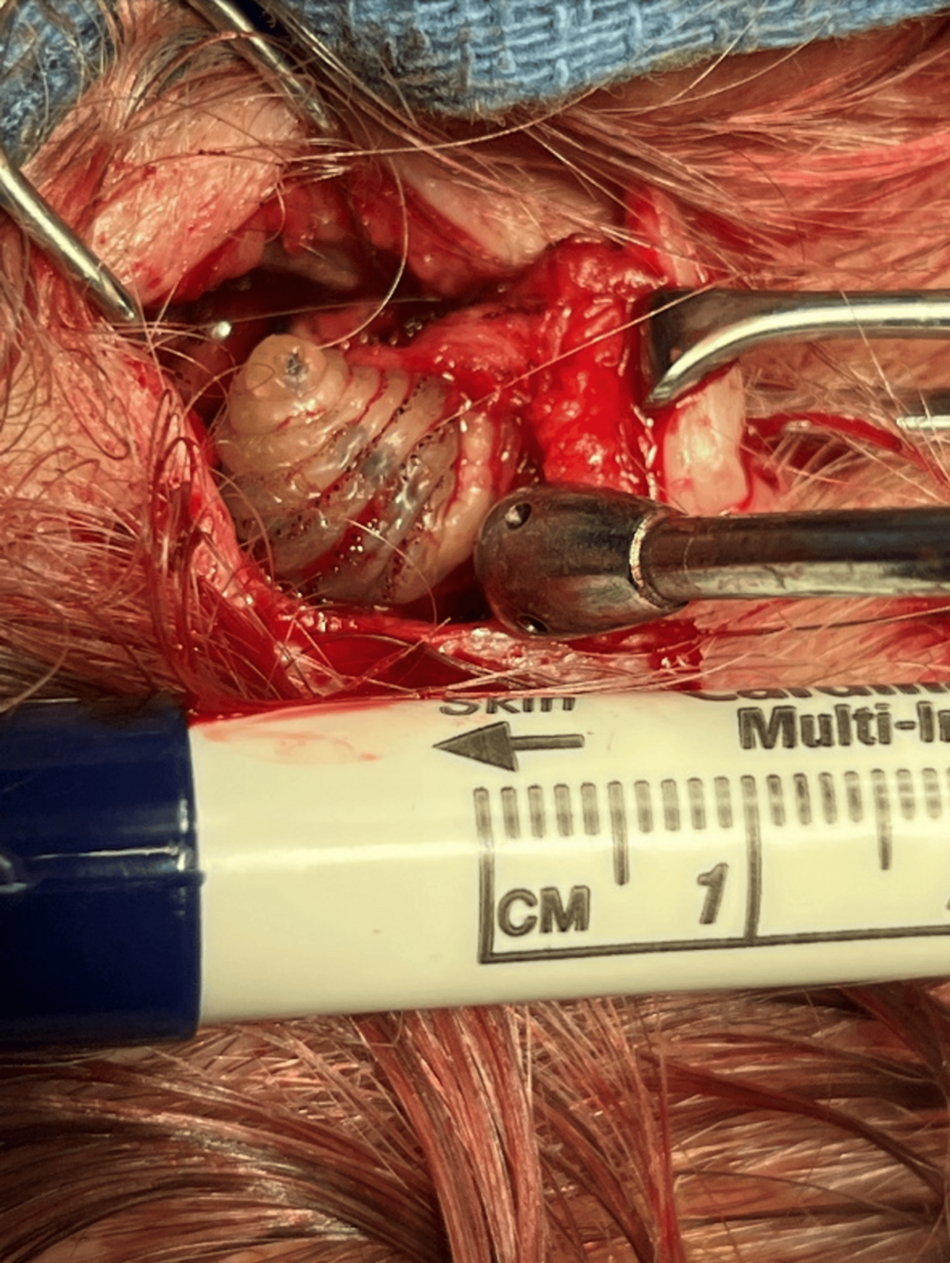
The embedded magot of Dermatobia hominis. Note the rows of spines and the tapered shape believed to prevent simple extrusion.

The patient was placed on amoxicillin/clavulanic acid 875 mg/125 mg oral tablet twice a day and cetirizine 10 mg oral tablet once a day for seven days. Failing to improve in one week, he was scheduled at an ambulatory surgery center to excise and repair a complex scalp wound under a local anesthetic 10 days after being originally seen. In the operating room, the area was sterilely prepped and infiltrated with local anesthesia, and a 1.5 cm x 0.5 cm ellipse of the scalp, including the furuncle, was incised with a scalpel. Upon dissecting around the scalp ellipse, it was determined that a moving, insect-like structure or maggot was within the subcutaneous tissue, attached to the furuncle, as shown in Figure 2. Careful dissection around the larva was performed, and it was removed in its entirety, alive, with some surrounding scalp tissue. 

**Figure 3:**
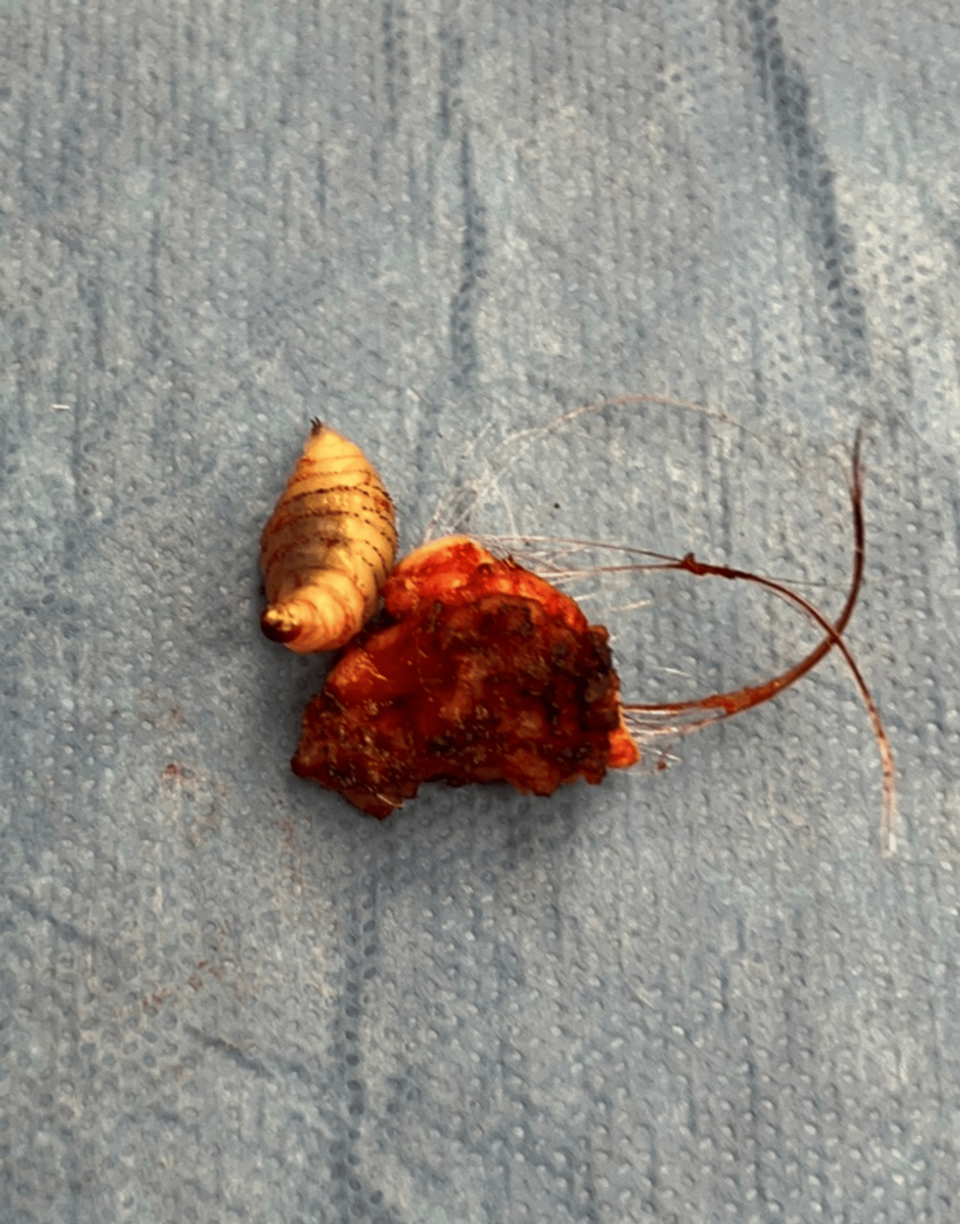
Maggot with excised scalp. This photo was utilized for Google Lens AI (Google LLC, Mountain View, United States), which revealed the suggested diagnosis.

The live parasite, as shown in Figure 3, was then photographed intraoperatively using a smartphone and Google Lens AI (Google LLC, Mountain View, United States), which immediately suggested the specimen was likely a larva of *Dermatobia hominis*. Google Lens is an AI image recognition software based on a neural network (the underlying technology in deep learning and AI), and it is ubiquitously present on mobile phones worldwide. In this case, it was accurate and easily verifiable due to the multitude of provided sources and comparable images. Dozens of similar photographs were presented with this AI-driven search, along with videos of live larvae extracted from their human hosts. Google AI was then used to rapidly gather peer-reviewed case reports of similar surgical extractions and safe curative therapies. The wound was debrided, copiously irrigated, and closed with a 4.0 synthetic, non-absorbable, monofilament suture. Postoperatively, the patient was informed of the suspected identity of the parasite and was reassured that the literature indicated no lasting effects. The larva was sent for pathological examination, which confirmed the identification of *D. hominis *larva nine days later. The incision healed without complications.

## Discussion

The life cycle of the human botfly is initiated by the female *D. hominis *fly, which attaches her eggs to the abdomen of a blood-sucking arthropod, usually a mosquito [1]. Once a human is bitten by the affected insect, the eggs of *D. hominis* hatch under the skin of the host, where the maggot feeds and grows in a subdermal cavity, breathing from a central punctum in the skin. Human botfly larvae rarely migrate far from the point of inoculation, unlike other botfly species. Belize, Bolivia, and Brazil have the highest infection rates of *D. hominis* in travelers. The larval stage can last from four to 18 weeks, until the larva emerges from the skin of the host and burrows into the soil or environment (pupa), and weeks later, emerges as an adult fly. The adult *D. hominis* form is rarely seen and is around 1-3 cm long. The whole life cycle of *D. hominis *lasts approximately 3-4 months. 

This case report demonstrates an innovative use of AI in assisting the surgeon with limited resources in the perioperative diagnosis of a human botfly infection. In the setting of a suburban or rural community-based ambulatory surgery center, immediate access to experts such as infectious disease specialists or pathologists can be limited. The universal availability of AI to anyone with internet access and a smartphone or camera in the United States is making this technology more ubiquitous and helpful, especially in diverse suburban or underserved rural community-based practices, including global healthcare environments. In rural medicine, specialists are often geographically distant, and physicians can find themselves in a position of performing procedures that may be outside their comfort zone to provide optimal patient care [4]. The burgeoning synthesis of medical imaging and AI over the past several years has resulted in significant advancements in healthcare delivery [5]. As such, we believe AI can help a diverse array of clinicians working in communities with limited resources.

Dermatologic diseases, which can affect up to 8-13% of all international travelers, are the third most common affliction among these travelers (diarrhea and respiratory illness are the most common). Sun exposure, contact allergies, and insect bites are included in this wide range of dermatologic conditions. Myiasis, the fourth most common travel-associated skin condition, is a parasitic infestation of vertebrate animal tissues caused by maggots of two-winged flies [6]. Cutaneous furuncular myiasis, caused by *D. hominis*, is endemic to many parts of Mexico, Central America, and South America [7]. In the United States, where myiasis is uncommon and unfamiliar to many clinicians, furuncular myiasis can mimic an allergic response to an insect bite, a persistent cutaneous abscess, an inflamed sebaceous cyst, or an unusual neoplasm [7]. Ultimately, the official diagnosis of the *D. hominis *infection in this case report was generated 10 days after the specimen was physically transported and processed at a regional pathology center. Gross analysis, combined with the clinical background given to the regional pathology experts, provided sufficient data to determine the diagnosis and support the surgeon’s suspicion. With Google Lens, the surgeon was provided timely and accurate diagnostic information to support his own clinical suspicion, which was further confirmed by the patient’s expanded history while undergoing the procedure under a local anesthetic.

The use of AI has shown significant promise in assisting clinicians in the early detection and diagnosis of skin diseases, including skin cancer. For instance, advanced AI systems can now analyze dermatological images with high precision, recognizing subtle patterns that even experienced dermatologists struggle to detect. Han et al. demonstrated that AI utilization of deep neural networks could be accurate in diagnosing benign and malignant skin lesions, at times outperforming experienced dermatologists [3]. Zare et al. recently developed an AI-based algorithm for the automated diagnosis of leishmaniasis using 300 microscopic images. One goal of these researchers was to enhance the global healthcare system and rural medicine’s capacity to speed up diagnoses and, therefore, facilitate effective treatment for leishmaniasis, one of the world’s most deadly parasitic diseases [5].

## Conclusions

We present the first case report of an AI-assisted intraoperative diagnosis of cutaneous furuncular myiasis. We hope that the awareness of our utilization of a common AI technology and the surgeon’s resourcefulness, demonstrated in this case report, can add to other clinicians’ “bag of tricks” in similar scenarios. Undoubtedly, research and academic centers will continue to play an important role in shepherding AI technology usage in healthcare. Clearly, future randomized controlled studies of images of cutaneous lesions using ubiquitous AI systems in a travel medicine clinic could help determine the accuracy of these technologies and develop real-world evaluations of AI systems in the diagnoses, treatments, benefits, and risks to patients and clinicians using this technology to support care. The successful use of this type of AI also depends on effective clinician-AI collaboration, with transparency of database input and continuous physician oversight. However, we believe that AI-assisted image analysis for disease diagnosis should not replace the physician. Currently, AI offers many community-based providers a means to better serve their diverse, often resource-limited patient populations. Physicians using AI can leverage the wealth of information contained within databases and medical images to provide accurate diagnoses, personalize treatment plans, and fundamentally improve patient outcomes. As AI technology continues to advance, we can expect even more groundbreaking innovations that will further transform the landscape of healthcare delivery. Physicians should strive to become adept at utilizing medical imaging and AI systems in the future to improve patient outcomes. 

Challenges in AI adoption in healthcare are numerous including clinician education and training, integration into existing workflows, and the potential resistance to relying on AI for diagnosis. Transparency and oversight of AI-assisted models will be necessary for more accurate, safe, and successful adoption of AI in healthcare. Post-deployment monitoring of these AI systems will be necessary for quality assurance purposes. By continuously learning from our colleagues’ experiences and ingenuity, we have come to appreciate that AI cannot take over the role of the clinician but can, at times, remarkably improve patient care. New breakthroughs in healthcare often arise from old, rare diseases being reexamined through the lens of innovative modern technologies. More research in the realm of patient outcomes while utilizing AI can help guide future developments in this rapidly changing field.

## References

[1] Barron Barron, M M (2024). Driving the future of health. September 10.

[2] Pinto-Coelho L (2023). How artificial intelligence is shaping medical imaging technology: a survey of innovations and applications. Bioengineering (Basel).

[3] Han SS, Moon IJ, Kim SH (2020). Assessment of deep neural networks for the diagnosis of benign and malignant skin neoplasms in comparison with dermatologists: a retrospective validation study. PLoS Med.

[4] Miller KK (2011). The clinical, professional, and social challenges of practicing rural medicine. Virtual Mentor.

[5] Zare M, Akbarialiabad H, Parsaei H (2022). A machine learning-based system for detecting leishmaniasis in microscopic images. BMC Infect Dis.

[6] Joyce MP (2002). Skin diseases of travelers. Prim Care.

[7] (2024). Myiasis. September.

